# Control Work

**Published:** 1898-02

**Authors:** 


### CONTROL WORK.

*Massachusetts.* The annual report of the Board of Cattle Commissioners has been transmitted to the Secretary of State. It is an extensive document, covering 150 pages of typewritten matter, and gives in detail the year's work of the board and various suggestions for continuing public and animal health.

After a list of the local inspectors in all the towns and cities of the State, the report recites the change inaugurated last spring, by which the annual inspection of cattle was made just before the stock went to pasture, with the instructions sent to inspectors, and the result of the work. It is believed that the inspection was made much more thorough by this change. The inspectors were ordered to complete their work by May 1st, and on May 12th those who had delayed were ordered to close up at once, and that no more cattle should be quarantined until another inspection was ordered, unless some one reported badly diseased animals to boards of health. Inspectors of the border-towns were also reminded of the regulations relative to the admission of cattle from without the State.

The report has a table giving the number of cattle assessed in each town, the number tested by tuberculin, the number condemned, the amount of compensation awarded, and the amount of State tax paid from each town, from which it appears that 210,801 cattle were assessed, 9991 were tested, 5246 were condemned, and $179,735.52 was awarded to owners. The per cent. of animals condemned of those considered suspicious by physical examination, 52.5, is substantially the same as in former years, and confirms the uncertainty of physical examination. In addition to the above, there were 254 cattle condemned on which warrants had not been settled, these amounts and other outstanding bills amounting to $10,712.40

The towns in which the larger amounts of money for condemned cattle were disbursed were: Acton, $4751.50; Andover, $1922; Ashby, $1043; Billerica, $5477.51; Bolton, $1353; Boston, $3543.80; Roxboro, $1418; Carlisle, $8697.50; Chelmsford, $4854; Concord, $3065; Conway, $1426; Dracut, $5869.50; Framingham, $1377; Gardner, $2109; Grafton, $1109.50; Granby, $1031.50; Greenfield, $2067.50; Groton, $1068.50;

Hardwick, $1159; Harvard, $2150.50; Haverhill, $1374; Hopkinton, $1107.50; Lanesboro, $1320; Leominster, $2363.50; Lexington, $3937.50; Lincoln, $3563; Littleton, $1192; Lowell, $1448.50; Maynard, $3977.50; Methuen, $1311; Natick, $1726.50; Newburyport, $1424,50; Newton, $1189; North Andover, $3166; Peabody, $1185; Princeton, $2952; Sterling, $1613.66; Stow, $2801; Sudbury, $14.285.18; Sutton, $1344.50; Waltham, $2440; Wayland, $2084.50; Westboro, $2227; Westford, $1018.50; Westminster, $1018.50; Weston, $3202; Westport, $1542; Worcester, $2690.25.

The financial statement of the board is as follows:

| | |
|---|---|
| Paid for 5275 tuberculous cattle | $179,867 52 |
| Paid for 160 cattle, no lesions | 5,581 04 |
| Quarantine expenses | 2,928 11 |
| Arbitration | 27 75 |
| Killing and burial expenses | 125 93 |
| Total | $188,530 35 |
| Average per head 5435 cattle | 34 12 |
| Commissioners' salaries | 7,283 00 |
| Agents' salaries | 13,561 54 |
| Clerks' and stenographers' salaries | 5,201 04 |
| Expenses, commissioners | 3,781 80 |
| Expenses, agents | 11,869 28 |
| Expenses, office | 3,154 56 |
| Expenses, laboratory and exp. work | 1,515 17 |
| Expenses, implements | 1,883 75 |
| Expenses, quarantine stations | 3,870 35 |
| Expenses, glanders, killing, and burying | 87 00 |
| Total | $52,207 49 |
| Total payments | 240,737 84 |

The receipts of the year were $5217.29, of which $5039.74 was for hides and carcasses.

The report says the work of animal inspection has resulted in the gradual reduction of the number of cases of generalized or advanced tuberculosis, as is shown by the records of the last three years.

Previous to the legislative act private veterinarians had reported tests as follows:

| | Tests. | Condemned. |
|---|---|---|
| Dr. W. E. Peterson | 1498 | 838 |
| Drs. Lyman and Osgood | 660 | 314 |
| Dr. J. F. Winchester | 11 | 9 |
| Dr. M. Bunker | 35 | 24 |
| Dr. G. N. Kinnell | 42 | 37 |
| Dr. G. N. Kinnell | 295 | 3 |

| | Tests. | Condemned. |
|---|---|---|
| Dr. C. S. Moon | 49 | 22 |
| Dr. J. H. Seale | 8 | 8 |
| Dr. A. H. Streeter | 18 | 9 |
| Dr. A. L. Crandall | 14 | 2 |
| Dr. A. S, Cleaves | 3 | 1 |
| Dr. C. A. Hamblet | 18 | 17 |
| Dr. W. S. Eaton | 5 | 4 |
| Dr. E. Knobel | 5 | 5 |
| Dr. J. H. Dutton | 40 | 11 |
| Dr. J. W. Robinson | 28 | 18 |
| Dr. S. O. Fowle | 1 | 1 |

The work of the inspecting agent, to see how owners were obeying the requirements relative to sanitary measures, showed as follows:

| | First visit. | Second visit. | Third visit. |
|---|---|---|---|
| Filthy stables | 11 | 4 | 2 |
| Unclean stables | 42 | 7 | 4 |
| Clean stables | 35 | 32 | 4 |
| Satisfactory stables | 52 | 33 | 3 |

In the work of thorough disinfection where tuberculous cattle had been removed, the agent reported as follows:

| | First visit. | Second visit. | Third visit. |
|---|---|---|---|
| Filthy stables | 397 | 43 | 3 |
| Unclean stables | 141 | 37 | 11 |
| Clean stables | 120 | 211 | 106 |
| Satisfactory stables | 59 | 234 | 26 |

Under the law for the inspection of slaughtered animals at licensed slaughter-houses and on the premises of owners, 188,391 cattle, 405,368 sheep, and 1,471,679 swine have been inspected, and 302 cattle, 2 sheep, and 105 swine were condemned.

The work of inspecting animals from without the State at the public markets at Brighton, Watertown, and Somerville was as follows:

| | |
|---|---|
| Cattle for beef | 346,889 |
| Sheep | 937,070 |
| Swine | 1,419,466 |
| Calves | 139,067 |
| Horses | 28,822 |
| Cattle released on certificate | 19,468 |
| Cattle tested at stations | 227 |
| Cattle released at stations | 199 |
| Cattle condemned at stations | 28 |
| Cattle condemned (actinomycosis) | 2 |

During the year 7068 cattle have been admitted to the State on special permit.

In the laboratory and experimental work several herds have been retested with tuberculin from time to time, in attempt to ascertain sources of reinfection and immunity through repeated tests, but the tables of results given are valueless.

An investigation has also been carried on, under the direction of Dr. Theobald Smith, to ascertain whether bovine and human tuberculous bacilli are identical. He concludes that as found in the sputum they are not identical, and that human sputum is not specially dangerous to cattle.

The report says that while bovines may not be susceptible to human bacilli from sputum, it by no means follows that humans are not susceptible to the bovine bacilli, but expresses the opinion that the latter danger has been greatly exaggerated.

The report argues that the present practice of condemning the meat of slightly diseased animals is extravagant and wasteful.

The payment of full compensation for condemned cattle is criticised.

The appearance of Texas fever in this State in August, through cattle brought from the State of New York, causing the death of thirty-five or more animals, is extensively treated.

A number of cases of actinomycosis during the year are reported among cattle, and the disease is fully described, with details of its recent appearance.

The year has 485 cases of glanders reported, of which 402 have been killed, 21 were released, and 2 are under observation. This is an increase of 101 reported over 1896, and of 61 cases killed.

The action of the Boston Board of Health in securing a legislative act putting glanders, farcy, and rabies under its control, and ignoring the cattle commission, is criticised as a precedent that may be mischievous and pernicious.

There were 19 dogs reported as afflicted with rabies during the year, and in addition an outbreak at South Hadley in a herd of cattle in August, resulting in the loss of several cows.

The report is accompanied by a report from Dr. Langdon Frothingham of his work in the examination of portions of organs suspected of the disease, including tuberculosis, actinomycosis, and glanders, a total of 137. There were besides 24 inoculations for glanders, and 5 of rabies. He also discusses the present practice of destroying all carcasses infected with tuberculosis, and suggests the use of the carcasses for food where the disease is clearly localized.

Dr. Theobald Smith's report of his work is also given in detail of much interest.

*National.* The Department of Agriculture announces the successful production of a curative serum for hog-cholera. It is announced that Congress will be asked to appropriate a large sum of money for its production and use. Experiments made with the serum are said to have proven successful in 82 per cent. of the animals treated. As the fatalities in many outbreaks reach this percentage, it promises more than any line of investigation yet pursued.

*Maryland.* 1. A well-regulated system of slaughter-houses is as necessary to the public health as is a well-regulated system of schools to the public education.

2. Every slaughter-house is a centre of disease for the surrounding country, spreading trichinosis, echinococcus disease, gid, wireworm, and other troubles caused by animal parasites, and tuberculosis, hog-cholera, swine-plague, and other bacterial diseases.

3. The important factors concerned in spreading these diseases are offal-feeding, drainage, rats, and dogs.

4. These diseases may be greatly held in check, and in some cases entirely eradicated, in two ways: First, by a reduction in the number of premises on which slaughtering is allowed, on which account it is urged as all-important that there be a segregation of the slaughter-houses, so that all the butchers of any given town will be compelled to do their killing in a common inclosed and restricted area. In abandoning slaughter-houses, care should be taken to destroy the rats, in order to prevent the spread of infection. Second, by regulating the factors concerned in spreading the diseases: (*a*) Offal-feeding should be abolished; (*b*) drainage should be improved; (*c*) rats should be destroyed; and (*d*) dogs should be excluded from slaughter-houses.

5. A licensing of slaughter-houses by the State boards of health and the employment of an assistant State veterinarian, whose sole or most important duty shall be a sanitary supervision of all places where animals are slaughtered for food, are necessary.

6. The appointment on every local board of health of a competent veterinarian, whose duty it shall be to control the class of meat placed upon the block, is urged. All meats should be inspected at the time of slaughter, thus securing for the local consumer the same guarantee that the National Government provides for the foreign consumer and for interstate trade.

7. The prohibiting of the raising of any kind of stock within the premises of slaughter-houses is advised, as are also State regulations to the effect that when a stock animal (horse, of course, excepted) once enters the premises of a slaughter-house it must never be allowed to leave those grounds alive, but must be slaughtered within two weeks' time.

8. In justice to butchers, and as a protection to the consumer, I strongly advocate the introduction of the German Freibank in connection with every municipal slaughter-house.—*Summary of Address of Dr. Charles W. Stiles before the Maryland Medical Society.*

